# Organic Passivation‐Enhanced Ferroelectricity in Perovskite Oxide Films

**DOI:** 10.1002/advs.202400174

**Published:** 2024-06-18

**Authors:** Hao Meng, Bingbing Chen, Xiuhong Dai, Jianxin Guo, Wenheng Li, Yuhua Bai, Xuan Chang, Xuning Zhang, Jingwei Chen, Qing Gao, Baoting Liu, Jianhui Chen

**Affiliations:** ^1^ Advanced Passivation Technology Lab College of Physics Science and Technology Hebei University Baoding 071002 China; ^2^ Province‐Ministry Co‐Construction Collaborative Innovation Center of Hebei Photovoltaic Technology College of Physics Science and Technology Hebei University Baoding 071002 China

**Keywords:** defect passivation, enhanced ferroelectricity, organic polymer, Pb (Zr_0.4_, Ti_0.6_) O_3_, perovskite oxide films

## Abstract

Perovskite oxides and organic–inorganic halide perovskite materials, with numerous fascinating features, have been subjected to extensive studies. Most of the properties of perovskite materials are dependence on their ferroelectricity that denoted by remanent polarization (*P*_r_). Thus, the increase of *P*_r_ in perovskite films is mainly an effort in material physics. At present, commonplace improvement schemes, i.e., controlling material crystallinity, and post‐annealing by using a high‐temperature process, are normally used. However, a simpler and temporal strategy for *P*_r_ improvement is always unavailable to perovskite material researchers. In this study, an organic coating layer, low‐temperature, and vacuum‐free strategy is proposed to improve the *P*_r_, directly increasing the *P*_r_ from 36 to 56 µC cm^−2^. Further study finds that the increased *P*_r_ originates from the suppression of the oxygen defects and Ti defects. This organic coating layer strategy for passivating the defects may open a new way for the preparation of higher‐performance and cost‐effective perovskite products, further improving its prospective for application in the electron devices field.

## Graphical abstract

An organic coating layer, direct, low‐temperature and vacuum‐free strategy to improve the remanent polarization (*P*_r_) of perovskite oxide films is employed. The *P*_r_ is significantly increased from 36 to 56 µC cm^−2^. This organic coating layer strategy may open a new way for exciting and transformative developments of perovskite materials, further improving its prospective for application in the electron devices field.

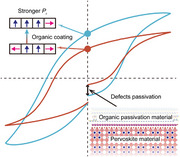

## 1 Introduction

Perovskite materials have numerous fascinating features. Notably inorganic perovskite oxides (Pb(Zr_x_, Ti_1‐x_)O_3_, BiFeO_3_, BaTiO_3_ SrRuO_3_) and metal halide perovskites (MAPbI_3_, FAPbI_3_, CsPbI_3_) are particularly interested by researchers.^[^ ^1^, ^2^ ^]^ Especially perovskite oxides, it has unique electrical,^[^ ^3^, ^4^ ^]^ thermal,^[^ ^5^ ^]^ optical^[^ ^6^ ^]^ and mechanical^[^ ^7^ ^]^ properties, which have excellent potential for applications in memory (ferroelectric random memory, non‐volatile ferroelectric memory),^[^ ^8^, ^9^ ^]^ sensors (piezoelectric sensors, photoelectric sensors, thermoelectric sensors),^[^ ^10^, ^11^ ^]^ piezoelectric transducers,^[^ ^7^, ^12^ ^]^ and anomalous photovoltaic effects.^[^ ^6^, ^13^ ^]^ These memory characteristics or anomalous photovoltaic properties of perovskite oxides were deeply dependent on its ferroelectricity.^[^ ^8^, ^13^, ^14^ ^]^ For example, the outstanding ferroelectricity can enable a higher open‐circuit voltage for perovskite oxide devices.^[^ ^13^ ^]^ Actually, the ferroelectricity of perovskite oxides originates from the polarization properties of perovskite materials and the remanent polarization (*P*_r_) is often used to represent the level of its polarization properties.^[^ ^4^, ^15^, ^16^ ^]^ Here, *P*_r_ denotes the polarization that persists in perovskite oxides when the applied electric field is reduced to zero.^[^ ^4^, ^17^ ^]^ Therefore, increasing the *P*_r_ of perovskite oxides is a key scientific issue in materials physics.

In recent years, numerous studies have focused on enhancing *P*_r_, including reducing the bulk defect within perovskite oxide crystals and suppressing interface defects.^[^ ^8^, ^18^, ^19^, ^20^, ^21^, ^22^, ^23^, ^24^, ^25^ ^]^ Optimization of preparation processes,^[^ ^21^, ^22^, ^23^, ^24^ ^]^ annealing conditions,^[^ ^25^ ^]^ doping techniques,^[^ ^19^ ^]^ and introduction of seed layers are employed for reducing bulk defects and improving *P*_r_.^[^ ^26^ ^]^ Controlling the lead/oxygen defects in Pb(Zr_x_, Ti_1‐x_)O_3_ (PZT) thin films by Mn‐doped, which improved the crystallinity and enhanced *P*_r_.^[^ ^19^ ^]^ Another research has shown that the *P*_r_ of the PZT films is tuned in the range of 6.6–42.2 µC cm^−2^, through controlling oxygen pressure change during pulsed laser deposition.^[^ ^24^ ^]^ For metal halide perovskites, molecular engineering is employed to enhance the *P*_r_ by introducing the halogen substitution on organic components, increasing the *P*_r_ to 23.3 µC cm^−2^.^[^ ^15^ ^]^ In addition, oxygen defects at the interface are also one of the main causes of reduced polarization properties.^[^ ^27^, ^28^ ^]^ Previous studies have demonstrated that interface oxygen defects cause the shift of titanium (Ti^4+^) to Ti defects (Ti^3+^) in PZT materials, thereby reducing the *P*_r_ and making devices fatigue.^[^ ^8^ ^]^ So, suppression of the interface oxygen defects becomes particularly important. Several strategies have garnered significant attention for controlling interfacial oxygen defects: 1) using conductive perovskite oxide films as bottom electrodes. Conductive perovskite oxide films, such as SrRuO_3_ (SRO) and LaxSr_1‐x_CoO_3_ (LSCO), are used as the bottom electrode in perovskite devices.^[^ ^29^, ^30^, ^31^ ^]^ This approach aids in reducing oxygen defects and promoting the epitaxial growth of PZT crystals.^[^ ^29^, ^30^, ^31^, ^32^, ^33^ ^]^ 2) insert diffusion barrier layers between the ferroelectric films and the electrode interface. The introduction of barrier layers between PZT and the electrode is an effective method for suppressing defects in PZT films.^[^ ^34^, ^35^ ^]^ These commonplace strategies offer a route to enhancing the performance of perovskite *P*_r_. However, the introduction of conducting perovskite oxide films or barrier layer methods often involves complex or costly processes, such as high‐temperature or high‐vacuum preparation technologies.^[^ ^29^, ^30^, ^31^, ^32^, ^33^, ^34^, ^35^ ^]^ It is essential to explore a new improvement strategy being cost‐effective and low‐temperature passivation techniques to passivate the surface defects.

In this work, we introduce a novel, straightforward organic coating layer passivation strategy to mitigate the defects in perovskite materials. Sulfonate molecules serve a dual role in neutralizing oxygen defects and facilitating the oxidation of Ti^3+^ to Ti^4+^, which effectively enhances the *P*_r_ of perovskite oxides. This simple organic coating layer passivation technique presents a promising strategy for the performance enhancement of perovskite materials.

## 2 Results and Discussion

**Figure 1:**
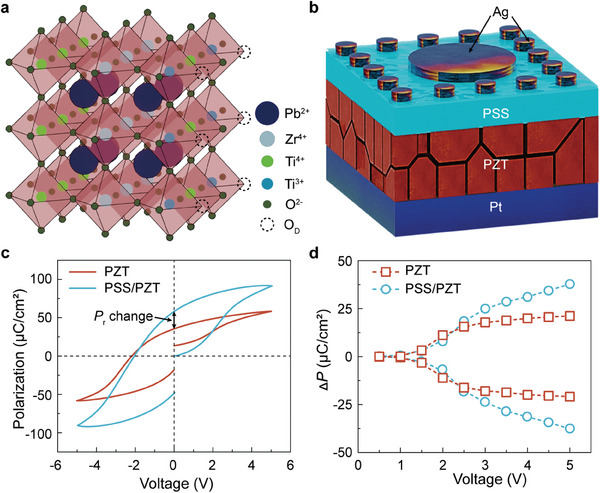
Structure of perovskite oxide materials and its characteristics. a) Schematic crystal structure of the PZT perovskite oxide; b) Schematic structure of Ag/PSS/PZT/Pt heterojunction; c) *P*–*V* loop of Ag/PSS/PZT/Pt and Ag/PZT/Pt capacitor; d) Switchable polarization of PSS/PZT and PZT capacitors with the applied voltage.

**Figure** **1**a illustrates the ABO_3_ perovskite structure of PZT, wherein “A” represents the lead (Pb), “B” denotes either zirconium (Zr^4+^) or Ti^4+^, and “O” includes oxygen (O).^[^ ^2^ ^]^ Typically, as‐grown PZT surfaces contain oxygen defects (O_D_) and Ti^3+^.^[^ ^36^, ^37^, ^38^ ^]^ In this context, the surface defects include B‐site defects (Ti^3+^), and oxygen defects. Here, the Ag/PZT/Pt heterojunction films with or without organic polymer polystyrene sulfonate (PSS) were fabricated on (111)‐oriented Pt/Ti/SiO_2_/Si substrates by sol–gel method. The PSS film was inserted between Ag and PZT interface, as presented in Figure 1b. The polarization–voltage (*P*–*V*) loop of both Ag/PZT/Pt (PZT) and Ag/PSS/PZT/Pt (PSS/PZT) capacitors at 5 V is shown in Figure 1c. The *P*_r_ of PZT and PSS/PZT capacitors are 36 and 56 µC cm^−2^, respectively. The *P*_r_ of the PSS/PZT increases by up to + 20 µC cm^−2^ from the as‐deposited after PSS treatment. As reported, the *P*_r_ may also be affected by the presence of charged defects.^[^ ^4^ ^]^ Subsequently, switchable polarization (Δ*P*) [Δ*P* = switched polarization (*P* ^*^) – non‐switched polarization (*P* ^^^)] is employed to further characteristic the *P*_r_ improvement of PZT capacitors with or without PSS film. Figure 1d presents the Δ*P* of PZT and PSS/PZT capacitors with the applied voltage (*V*).^[^ ^34^ ^]^ Notably, compared to the PZT capacitor, the PSS/PZT capacitor demonstrates an impressive 16 µC cm^−2^ increase in Δ*P* when the applied voltage of 5 V. The increased Δ*P* further confirms the positive effect of PSS on ferroelectricity. Figure S1 (Supporting Information) displays the statistical result of *P*_r_ with or without the organic coating layer at the applied voltage of 5 V. It clearly demonstrates that *P*_r_ of the most of PSS/PZT capacitors exceeds 50 µC cm^−2^, while the *P*_r_ of PZT capacitors is below 40 µC cm^−2^. This provides further evidence that PSS enhances the *P*_r_ of PZT devices.

To find out the reason for the increase of *P*_r_, we first suspect that the *P*_r_ may potentially influence the shared voltage of PZT by coating the PSS dielectric layer. Previous work has reported that PSS with a bandgap of 5.0 eV is a non‐ferroelectric dielectric material.^[^ ^39^, ^40^, ^41^ ^]^ After coating PSS, the applied external voltage will divide between PSS and PZT, and the relationship between their voltage division is shown in Equation (1) and (2).^[^ ^42^, ^43^, ^44^ ^]^

(1) $$V_{PSS}=\frac{V\varepsilon_{FE}}{d_{PSS}\varepsilon_{FE}+d_{FE}\varepsilon_{PSS}}$$

(2) $$V_{FE}=\frac{V\varepsilon_{PSS}}{d_{FE}\varepsilon_{PSS}+d_{PSS}\varepsilon_{FE}}$$

where *d*_PSS_ represents PSS film thicknesses, *d*_FE_ represents PZT film thicknesses, *ε*_PSS_ represents the PSS dielectric constant, and *ε*_FE_ represents PZT dielectric constant. Five PSS/PZT capacitors with different thicknesses of PSS film (ranging from 0 to 800 nm) were designed to investigate the possible influence of voltage division of PZT. It can be clearly seen that *V*_FE_ should theoretically decrease as *d*_PSS_ increases, leading to the decrease of Δ*P*.

**Figure 2:**
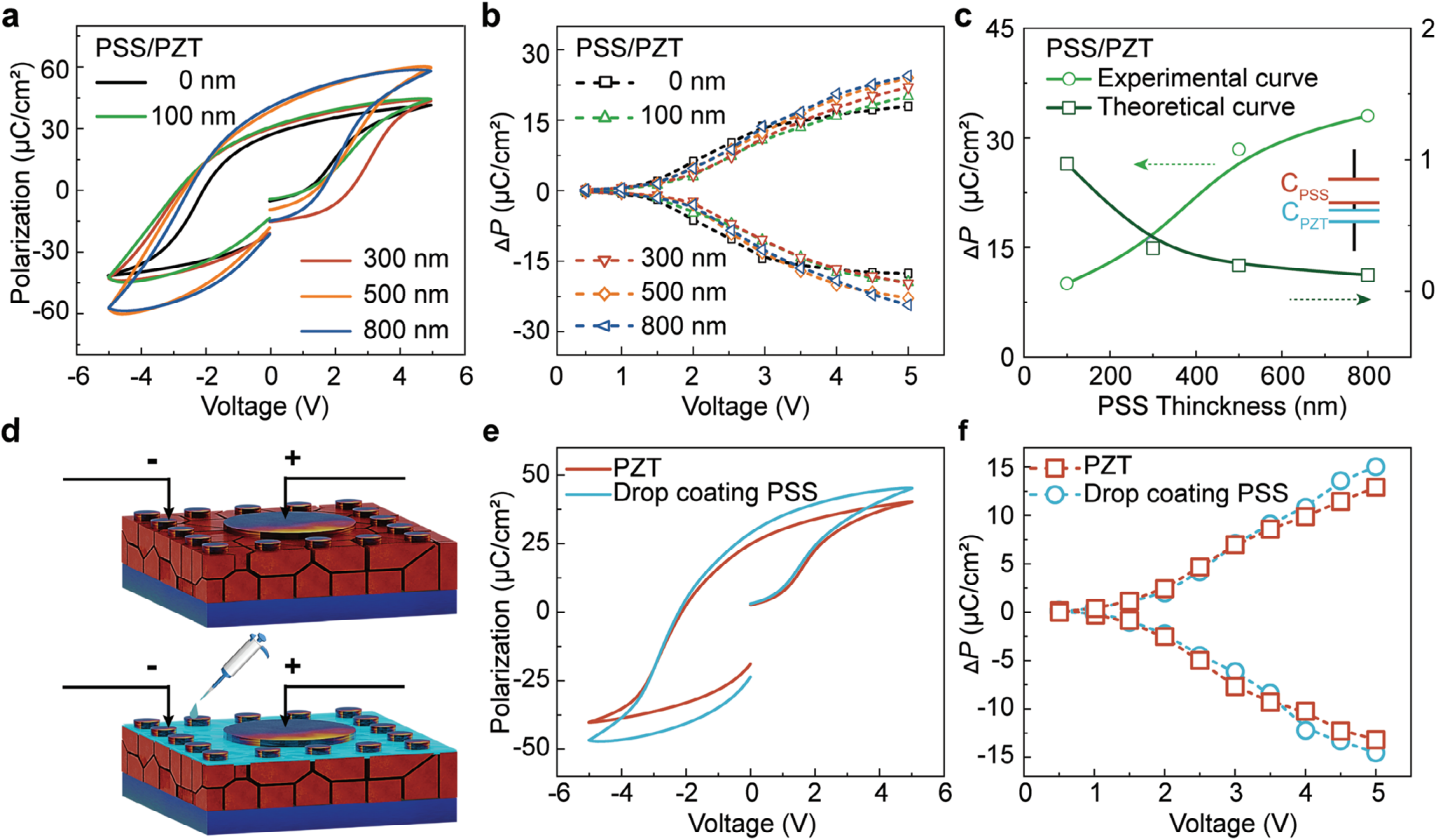
The possible reason for the increased ferroelectricity. a) *P*–*V* loop of PSS/PZT with varying PSS thicknesses; b) Switchable polarization with the applied voltage for different PSS thicknesses; c) Difference in switchable polarization of PSS/PZT versus PSS film thickness; d) Schematic of in situ drop coating of PSS; e) *P*–*V* loop with in situ drop coating of PSS; f) In situ drop coating of PSS: switchable polarization with the applied voltage.

In the actual experiment, the V_FE_ of the PZT in the PSS/PZT structure is very difficult be measure directly. Here, we use the change Δ*P* of to show the experiment trend of V_FE_. The *P*–*V* loops of PSS/PZT capacitors are shown in **Figure** **2**a. The study clearly exposed that the *P*_r_ value of PSS/PZT improved with the increase in PSS thickness. Moreover, the Δ*P* of the five PSS/PZT capacitors was also measured and is presented in Figure 2b. The experimental curve of Δ*P* which is derived from the data depicted in Figure 2b shows the completely opposite of the theoretical calculation. Therefore, the enhanced *P*_r_ of PSS/PZT has nothing to do with the shared voltage by the PSS dielectric layer.

To gain further insight into the independence of the enhanced *P*_r_ with the dielectricity of PSS, an in situ drop coating of the PSS solution is implemented on the PZT surface. The schematic diagram of the in situ drop coating is illustrated in Figure 2d. The *P*–*V* loop and Δ*P* are presented in Figure 2e,f, respectively. During this in situ passivation test, PSS only passivates the defects around the electrode, whereas the normal coating (Figure 1b) can fully passivate the surface defects of PZT. Therefore, the enhanced Pr observed is much less effective compared to normal coating. It can be found that the *P*_r_ of the PZT capacitor is also enhanced by the in situ drop coating PSS. The results further confirm that the PSS increases the *P*_r_ and it is not related to the dielectricity of PSS.

**Figure 3:**
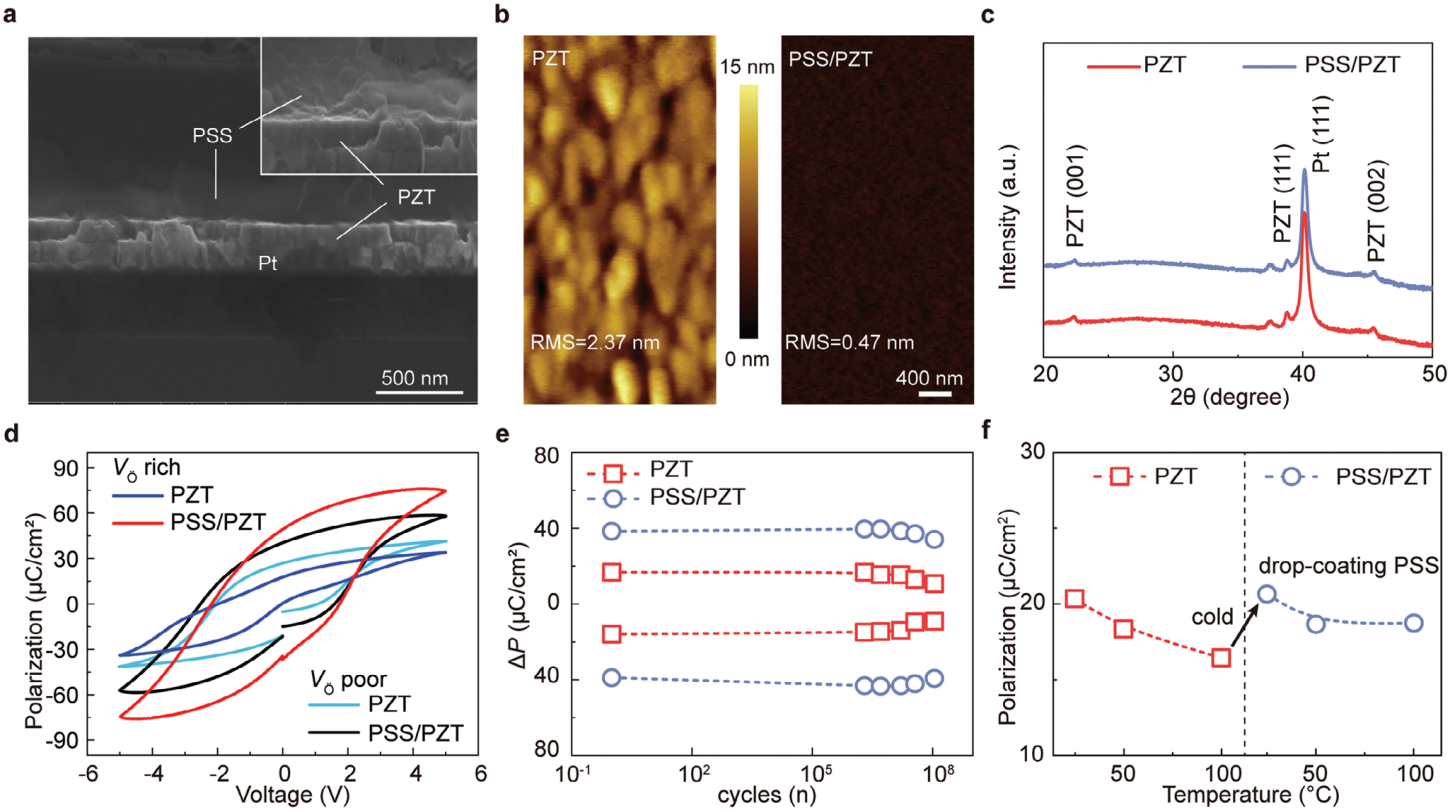
a) Cross‐sectional images of PSS/PZT film measures by SEM; b) AFM images of PZT and PSS/PZT films; c) XRD spectrum of the PZT and PSS/PZT heterostructure films; d) *P*–*V* Loop of PZT under oxygen‐poor and oxygen‐rich conditions; e) fatigue endurance of PZT capacitors and PSS/PZT capacitors; f) thermal stability of PZT capacitors and PSS/PZT capacitors.

The cross‐sectional PSS/PZT analysis is performed using scanning electron microscopy (SEM). As shown in **Figure** **3**a, PSS film is deposited uniformly on the PZT surface. The larger magnification SEM image shows that a high‐quality coverage is formed between PSS and PZT without any pinholes. As shown in Figure S2a,b (Supporting Information), there are pinholes on the surface of PZT. However, as illustrated in Figure S2c,d (Supporting Information), the surface of PSS/PZT coated with PSS does not show any pinholes. This confirms the significant effect of the PSS on surface morphology. Additionally, the atomic force microscopy (AFM) image is also employed to assess the surface morphology of PZT and PSS/PZT films (Figure 3b). The value of root‐mean‐square (RMS) is 2.37 nm for PZT film, which decreases to 0.47 nm upon coating PSS on the PZT surface. The surface roughness of the PSS/PZT is notably lower than that of the PZT film. The AFM result further emphasizes the uniform distribution of PSS on the surface of PZT, highlighting the significant impact of the surface morphology‐modifying influence. Subsequently, we conducted X‐ray diffraction (XRD) test on PSS/PZT and PZT films (Figure 3c). The lattice structure of PSS/PZT and PZT films is identical, thus negating the possibility of any changes induced in the lattice and crystallinity of PZT by the PSS surface coating.

Up to here, we clearly demonstrated that the enhancement *P*_r_ is independent of the dielectricity of PSS and not impact the lattice structure of PZT, while the surface morphology of PSS/PZT films has changed. From our previous work, PSS with an outstanding passivation capability for silicon and Cu(In, Ga)Se_2_ surface defects, so we wondered if it could passivate the surface defects of PZT.^[^ ^39^, ^40^, ^45^ ^]^ Therefore, two types of PZT films with different oxygen vacancies (a species of oxygen defects, *V*_ö_) concentrations (*V*_ö_ poor and *V*_ö_ rich) are designed. As shown in Figure 3d, for both *V*_ö_ rich and *V*_ö_ poor films, the *P*_r_ of the films is significantly enhanced by PSS treatment. Notably, compared to *V*_ö_ poor films, the *P*_r_ enhancement is more obviously increased in *V*_ö_ rich films, which may affirm the function of PSS in passivating oxygen defects. Figure 3e illustrates the fatigue characteristics of both PSS/PZT and PZT, revealing a noticeable attenuation in polarization decay for PSS/PZT compared to PZT. The results further confirm that PSS can passivate oxygen defects of PZT. Nevertheless, influenced by the interface between PZT and the bottom electrode (Pt), PSS/PZT still exhibits fatigue phenomena. Furthermore, Figure 3f shows the thermal stability of PZT with in situ drop coating of PSS, where it is evident that PSS enhanced PZT of polarization. The PZT film coated with PSS exhibits comparable thermal stability, maintaining relative stability even in a 100 °C environment.^[^ ^44^ ^]^

**Figure 4:**
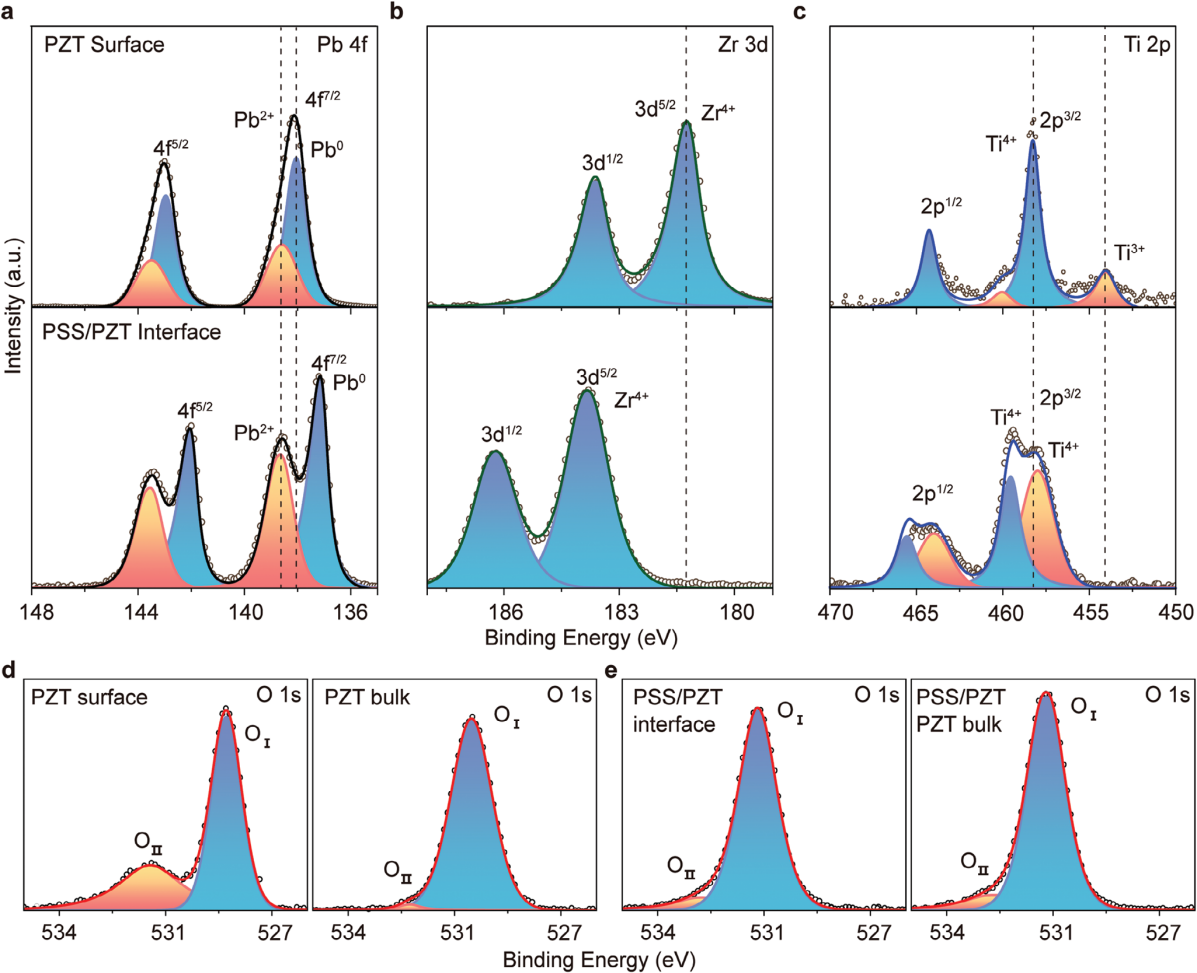
XPS spectra of PZT surface and PSS/PZT interface. a) Pb 4f spectrum; b) Zr 3d spectrum; c) Ti 2p spectrum; d) O 1s at different etching times of PZT film. e) O 1s at different etching times of PSS/PZT film.

To further investigate the passivation effect of PSS, X‐ray Photoelectron Spectroscopy (XPS) was applied to analyze the elemental composition of both the PZT surface and the interface between PSS and PZT. As illustrated in **Figure** **4**a–e, the XPS spectrum of the elements Pb^2+^, Zr^4+^, Ti^4+^, and O^2−^ on the PZT and PSS/PZT films are consistent with the prior studies.^[^ ^46^, ^47^ ^]^ Figure S3a (Supporting Information) illustrates the atomic ratio of PZT concerning the etching time. As the etch time increases, there is a significant decrease in the content of Pb elemental and a significant increase in the O elemental content, indicating the presence of oxygen defects and the Pb^0^ on the surface of the PZT film, which are consistent with other reports.^[^ ^23^, ^36^, ^48^ ^]^ Figure S3b (Supporting Information) presents the atomic ratios of Pb, Zr, Ti, O, and S elements in the PSS/PZT heterojunction at different etch times. When the etching time is 1000 s, the increased Pb and decreased S elements denote the appearance of the interface for PSS and PZT.

As shown in Figure 4a, for the Pb 4f spectrum of pristine PZT, discernible binding energies (BE) at 137.2, 138, 138.6, and 138.7 eV are attributed to Pb^0^ and Pb^2+^. Surprisingly, a peak area ratio of Pb^2+^ and Pb^0^ increased from 0.36 to 0.46, as well as the Pb^0^ peak is shifted from 138.0 to 137.2 eV at the PSS/PZT interface. Figure S4 (Supporting Information) further elucidates that the shifted peaks and increased Pb^0^ at the PSS/PZT interface are associated with the etching process.^[^ ^49^ ^]^ For the Zr 3d XPS spectrum, the BE at 181.2 and 183.8 eV is assigned to Zr^4+^ (Figure 4b). Notably, compared to the PZT surface, the Zr^4+^ peak at the PSS/PZT interface shows an elevation of 2.6 eV. The BE shift of zirconium is also affected by etching which is the same as the previous report.^[^ ^49^ ^]^ For the Ti 2p XPS spectrum, BE values at 454.0, 457.9, 458.3, and 459.5 eV, correspond to Ti^3+^ and Ti^4+^ (Figure 4c). The presence of the Ti^3+^ peak at 454.0 eV is indicative of oxygen defects, and this finding is consistent with the prior studies. However, according to the stoichiometry of PZT Ti^4+^ is necessary for a good ferroelectric property of PZT.^[^ ^8^, ^27^ ^]^ After introducing PSS at the PZT surface, the Ti^3+^ peak at 454.0 eV is shifted to the Ti^4+^ peak at 457.9 eV, demonstrating PSS facilitates the oxidation of reduced Ti^3+^ ions to Ti^4+^ ions.^[^ ^50^ ^]^ The Ti^4+^ peak at 459.5 eV is related to the 458.3 eV Ti^4+^ peak affected by etching, as illustrated in Figure S4c (Supporting Information). The absent Ti^3+^ peak and newly occurred Ti^4+^ confirmed the surface passivation effect of PSS. To further verify the passivation function of PSS film, the O 1s XPS spectra in Figure 4d,e are employed. The O1s XPS spectra of the PZT and PSS/PZT exhibited single‐lobed peaks, which were asymmetric indicating that there are different types of oxygen existing on the PZT and PSS/PZT. Each peak could be fitted with two peaks (O_I_ and O_II_) representing two different kinds of surface species (Figure 4d,e). O_I_ with a low BE peak is characteristic of lattice oxygen, while O_II_ with a high BE peak is characteristic of non‐lattice oxygen (adsorbed oxygen or hydroxyl‐like group, etc.).^[^ ^50^, ^51^, ^52^, ^53^, ^54^, ^55^, ^56^, ^57^ ^]^ Figure 4d shows a notable difference in the intensity of O_II_ peak between the surface and bulk of PZT. In Figure S3a (Supporting Information), the oxygen elements within the PZT bulk are closer to normal stoichiometry. Those results indicate the abundant presence of non‐lattice oxygen on the surface of PZT. After etching, the adsorbed oxygen and hydroxyl groups are removed, thereby revealing more lattice oxygen. As reported, the O_II_ peak is detrimental to the ferroelectric properties of PZT, reducing the intensity of the O_II_ peak has a positive impact on the ferroelectricity of PZT.^[^ ^58^ ^]^ Comparing the O_II_ peaks of the PZT surface (Figure 4d) with the PSS/PZT interface (Figure 4e), it shows that the O_II_ peak of PSS/PZT is significantly reduced by coating PSS. Furthermore, the oxygen atomic ratio between the surface and bulk of PZT in Figure S3a,b (Supporting Information), it is observed that the percentage decreases from 15.17% to 7.41% after PSS coating. These results provide evidence that the passivation effect of PSS on the elimination of non‐lattice oxygen in PZT, is consistent with the passivation effect of PSS in Figure 3d. The observed oxygen peak shift in Figure 4d,e is attributed to the etching process.^[^ ^49^ ^]^ As previous reports, the passivation effect of PSS is attributed to its sulfonic acid group.^[^ ^40^, ^59^, ^60^ ^]^ To confirm the contribution of the sulfonic acid group of PSS, we develop a coating of sodium polystyrene sulfonate (SPS) on PZT. Figure S5a (Supporting Information) highlights the distinction between SPS and PSS. For SPS, the hydrogen atoms in the sulfonic acid group of PSS are replaced by sodium elements. As illustrated in Figure S5b,c (Supporting Information), SPS does not exhibit the *P*_r_ enhancement effect. The results highlight the key function of the sulfonic acid group in the enhancement of the *P*_r_ of PZT. So, PSS passivating the PZT surface defects is derived from the sulfonic acid group. This organic coating layer passivation strategy may also have the potential applicability in organic–inorganic halide perovskite.^[^ ^59^, ^60^ ^]^

## 3 Conclusion

In conclusion, this study presents a simple and effective organic coating layer passivation strategy to enhance the *P*_r_ of perovskite oxides. The organic PSS film efficiently passivates the oxygen defects and significantly oxidizes Ti^3+^ to Ti^4+^ at the PZT surface. The *P*_r_ of the PSS/PZT increases by up to +20 µC cm^−2^ from the as‐deposited after PSS treatment. This novel approach not only advances the fundamental understanding of ferroelectricity in perovskite oxide materials but also holds significant potential for other perovskite materials. The organic coating layer strategy passivated the defects, poising for exciting and transformative developments of perovskite materials in the future.

## 4 Experimental Section

### PSS/PZT Capacitors

The PbZr_0.4_Ti_0.6_O_3_ precursor solution (Mitsubishi Materials Corporation) was spin‐coated onto a Pt/Ti/SiO_2_/Si substrate at 4000 rpm for 40 s. Subsequently, the film was heat‐treated in an air environment at 350 °C for 5 min. The process was repeated three times to obtain amorphous PZT films with a thickness of ≈120 nm. The PZT/Pt heterojunction was further annealed at 550 °C for 1 h in the 10^5^ Pa oxygen (*V*ö poor) or 10^4^ Pa air (*V*ö rich) using a tube annealer. Afterward, the pristine PSS solution (Sigma–Aldrich, 18 wt% in H_2_O) served as the precursor solution and was spin‐coated onto the surface of the PZT film at 3500 rpm for 40 s. After coating, the PSS film was dried at room temperature for 10 min. Finally, vacuum thermal evaporation deposited Ag was used as the top electrode of Ag/PZT/Pt or Ag/PSS/PZT/Pt capacitors.

### Characterization

The ferroelectric behavior (*P*–*V* loops, Δ*P*–V) of the PSS/PZT and PZT parallel plate capacitors were measured using a ferroelectric tester (Radiant Technologies, Precision Multiferroic II and Ferroelectric Test System). The surface morphologies of the samples were observed by AFM (Oxford Instruments MFP‐3D) and SEM (Nova Nano SEM450, FEI). The structures of PSS/PZT and PZT thin films were characterized by XRD (Bruker AXS GmbH D8 Advance). The surface analysis was performed by XPS (Thermo Fisher Scientific ESCALAB 250Xi) in ultrahigh‐vacuum conditions (base pressure of 1 × 10^−9^ mbar) using an Al Kα x‐ray source (15 kV, 10 mA, 500 µm spot size).

## Conflict of Interest

The authors declare no conflict of interest.

## Supporting information

Supporting Information

## Data Availability

Research data are not shared.
