# Supplementary material for: Organic Passivation‐Enhanced Ferroelectricity in Perovskite Oxide Films
Source: Adv Sci (Weinh). 2024 Jun 18;11(31):2400174. doi: 10.1002/advs.202400174 (PMC11336970; doi:10.1002/advs.202400174)
Supplement: Supplementary file 1 — Supporting Information [file ADVS-11-2400174-s001.docx]

**Supporting Information**

**ORGANIC PASSIVATION-ENHANCED FERROELECTRICITY IN PEROVSKITE OXIDE FILMS**

*Hao Meng^1^, Bingbing Chen^1*^, Wenheng Li^1^. Yuhua Bai^1^, Xuan Chang^1^, Xuning Zhang^1^, Jingwei Chen^1^, Qing Gao^1^, Jianxin Guo^1^, Xiuhong Dai^1^, Shufang Wang^1^, Baoting Liu^1^ and Jianhui Chen^1^**

*^1^Advanced Passivation Technology Lab, College of Physics Science and Technology, Hebei University, Baoding 071002, China.*

*^2^**Province-Ministry Co-Construction Collaborative Innovation Center of Hebei Photovoltaic Technology, College of Physics Science and Technology, Hebei University, Baoding 071002, China.*

*Corresponding Author: [chenbingbing0219@](mailto:chenbingbing0219@)hbu.edu.cn (B. Chen); [chenjianhui@hbu.edu.cn](mailto:chenjianhui@hbu.edu.cn)(J. Chen).


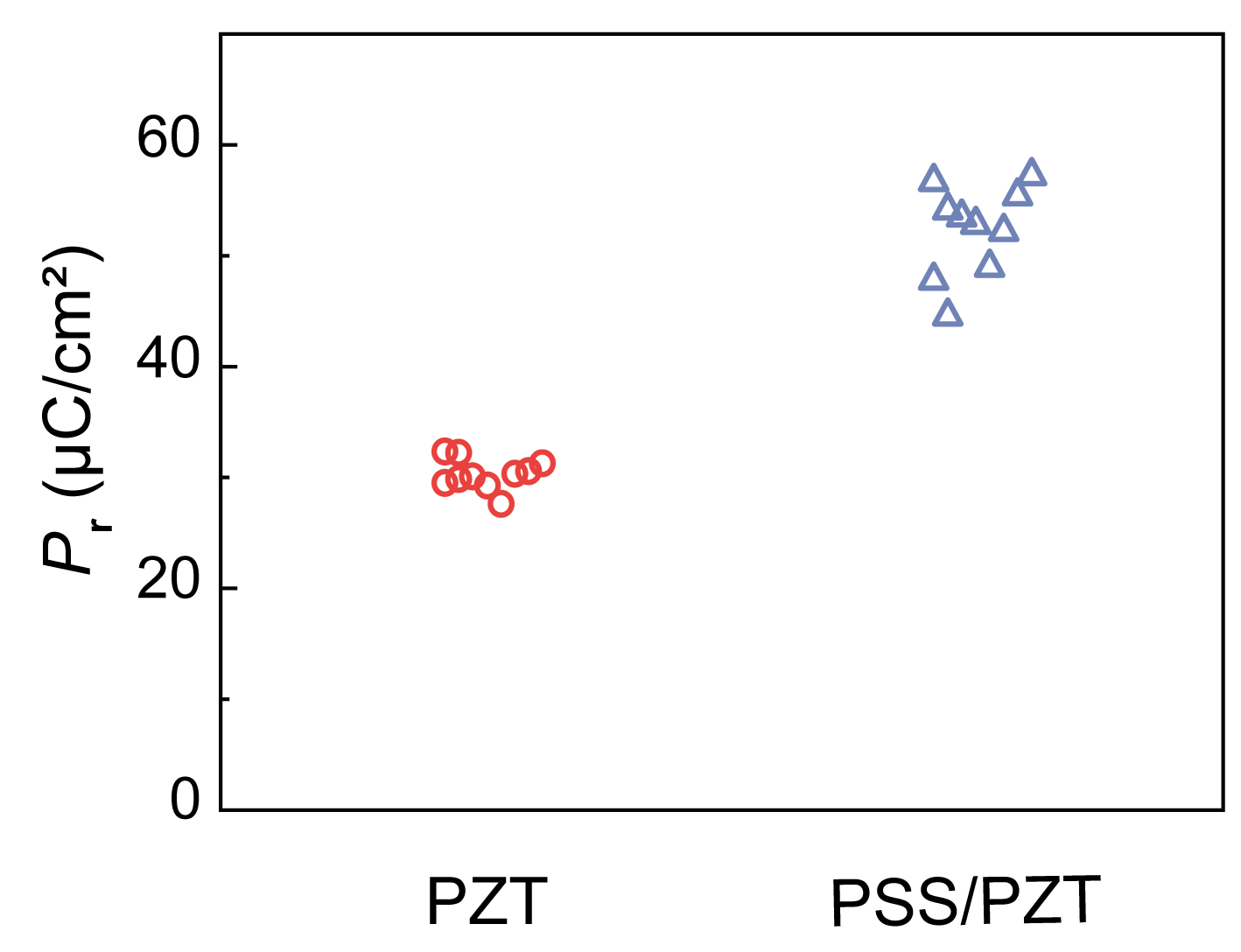


**Figure S1.** *P*_r_ comparison of PZT capacitors and PSS/PZT capacitors.


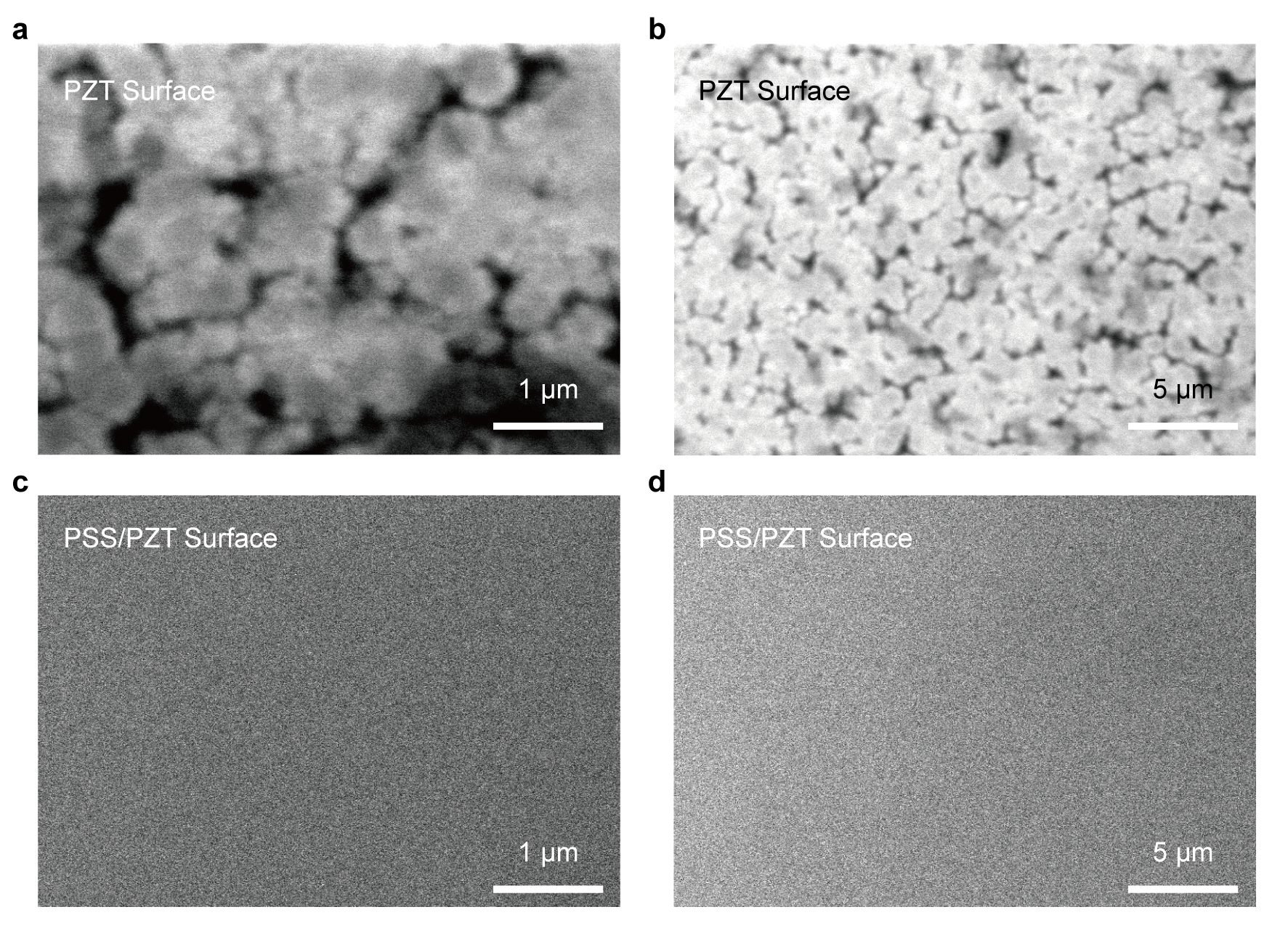


**Figure S2. Surface images of PZT and PSS/PZT films measures by SEM** (a), (b) PZT images with the scalebar of 1 and 5μm, respectively; (c), (d) PSS/PZT images with the scalebar of 1 and 5μm, respectively.


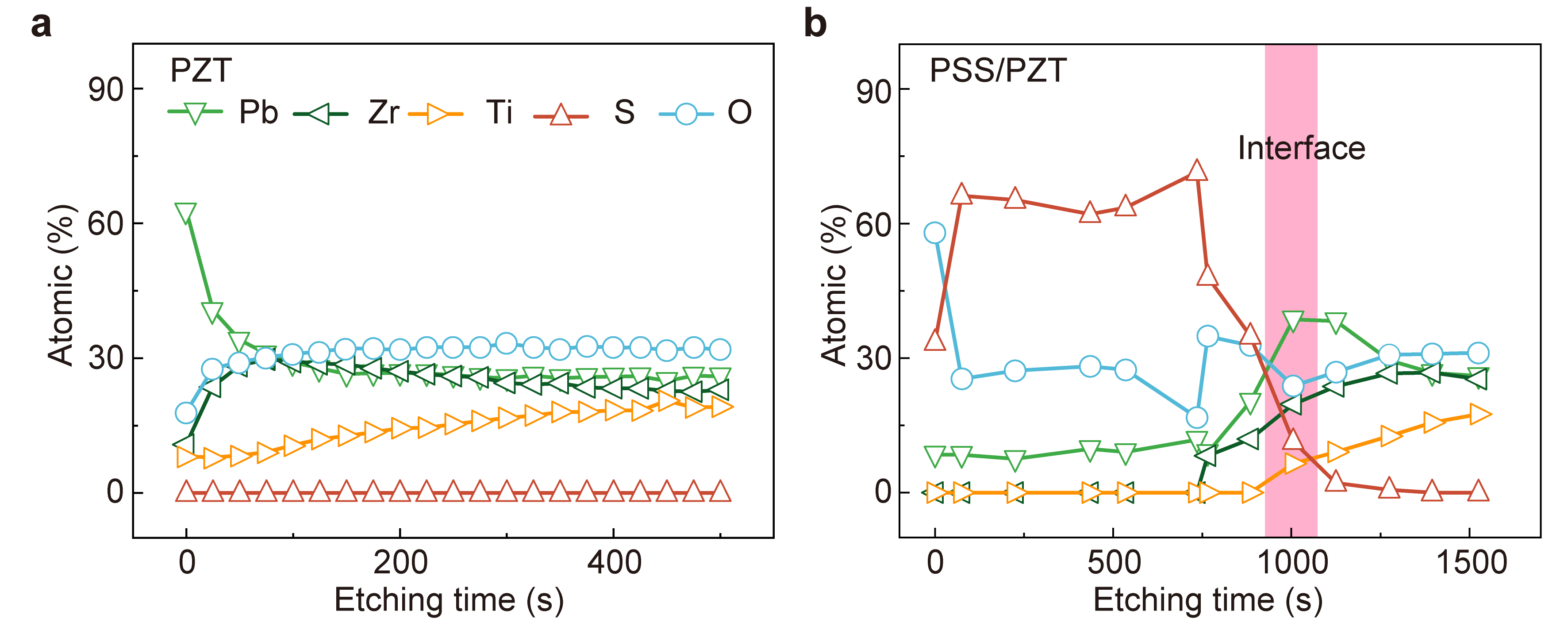


**Figure S3. Analysis of XPS etching** (a) Atomic ratio as a function of etching time in PZT; (b) Atomic ratio as a function of etching time in PSS/PZT.


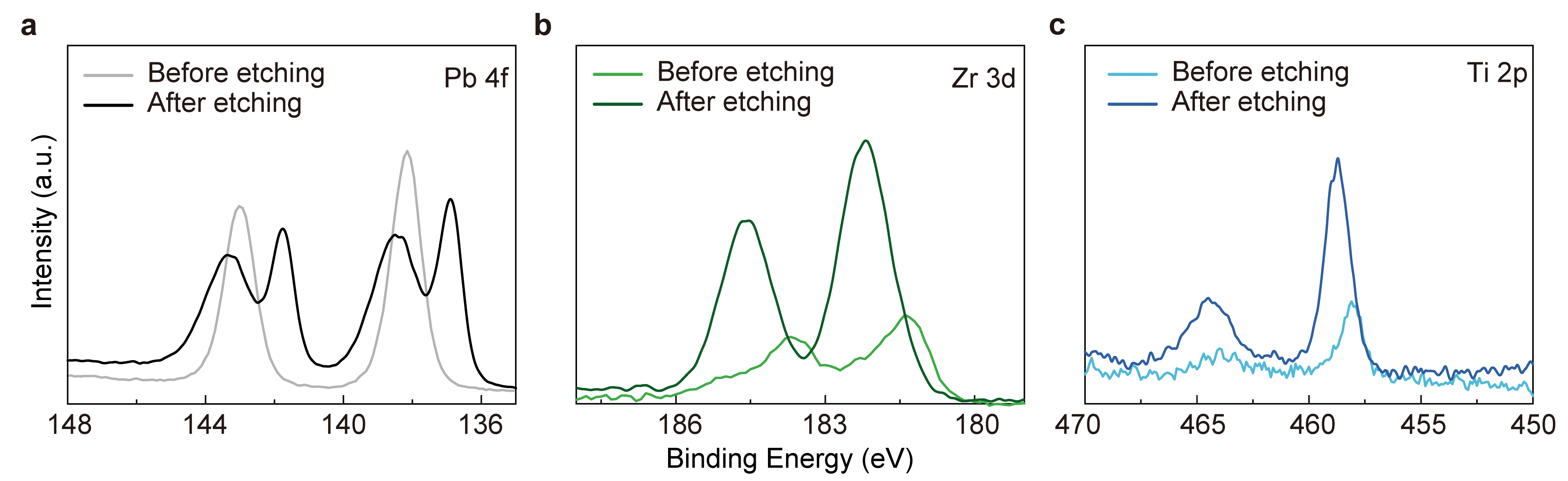


**Figure S4. PZT capacitors affected by Ar ion etching** (a) Pb 4f, (b) Zr 3d, (c) Ti 2p.


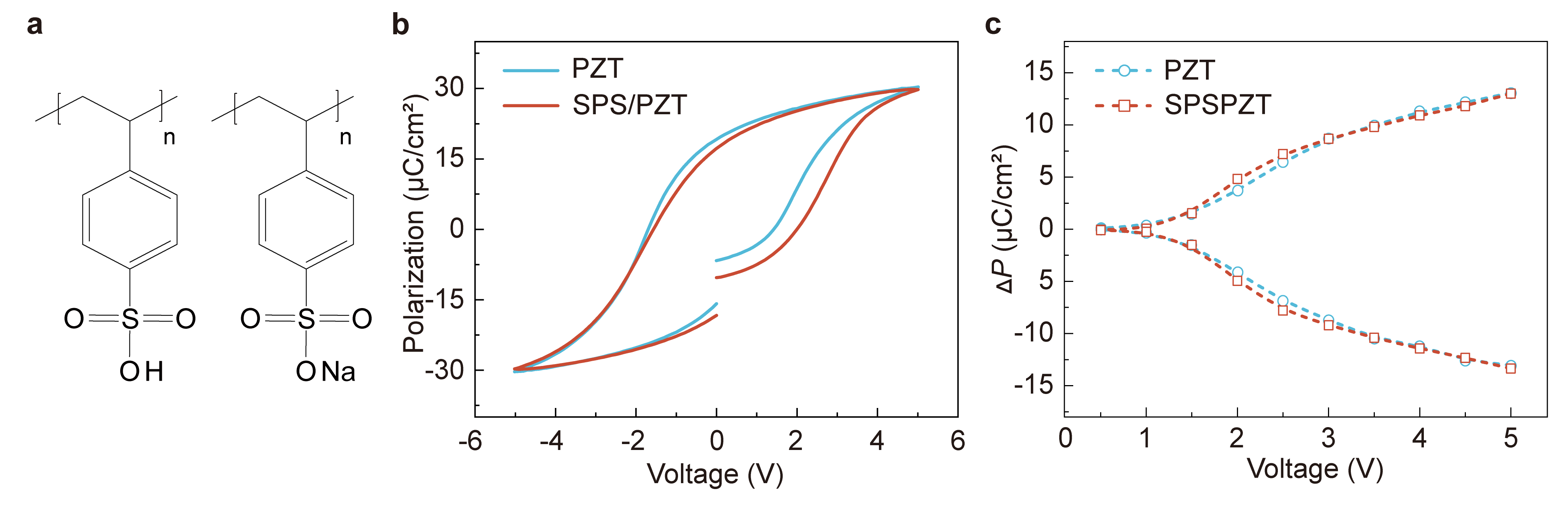


**Figure S5.** (a) Molecular formula of PSS and SPS; (b) P-V Loop of PZT and SPS/PZT capacitors; (c) Switchable polarization of PZT and SPS/PZT capacitors with the applied voltage.
